# Mortality from Parkinson’s disease and other causes among a workforce manufacturing paraquat: an updated retrospective cohort study

**DOI:** 10.1186/s12995-021-00309-z

**Published:** 2021-05-27

**Authors:** John Andrew Tomenson, Clive Campbell

**Affiliations:** 1 Causation Ltd, 2 Field View Drive, Macclesfield, Cheshire, SK11 7JN UK; 2 Syngenta Crop Protection AG, Basel, Switzerland

**Keywords:** Parkinson’s disease, Paraquat, Cohort study, Mortality study, Pesticides

## Abstract

**Background:**

Epidemiological studies of the association between Parkinson’s disease (PD) and paraquat (PQ) exposure have given inconsistent findings. The aim of the study was to update information on the risk of PD and mortality from major causes of death among a UK workforce who manufactured PQ by extending the follow-up by seven and a half years.

**Methods:**

This retrospective cohort study included all employees who had ever worked on any of the four plants at Widnes, UK where PQ was manufactured between 1961 and 1995. The 926 male and 42 female workers were followed through 31 December 2017. Mortalities for males were compared with national and local rates, including rates for PD as a mentioned cause of death.

**Results:**

A total of 394 male and 21 female workers had died by end of follow-up. Four death certificates of male workers mentioned PD, including two deaths that were due to PD. At least 6 death certificates of male employees would have been expected to have mentioned PD (SMR = 0.67; 95% CI 0.18–1.72). Reduced mortalities compared with local rates were found for major causes of death.

**Conclusions:**

The study provided no evidence of an increased risk of PD, or increased mortalities from other causes among PQ production workers whose exposure to PQ on a daily basis was at least comparable to that of a PQ sprayer or mixer/loader.

**Supplementary Information:**

The online version contains supplementary material available at 10.1186/s12995-021-00309-z.

## Background

Parkinsonism describes conditions that cause a combination of the movement abnormalities seen in Parkinson’s disease (PD), especially those resulting from the loss of dopamine-containing nerve cells (neurons). A specific cause is known for some forms of parkinsonism including vascular injury, repeated head injury, exposure to toxins such as manganese, and neuroleptic medications. However, a vast literature on lifestyle and environmental possible risk or protective factors exists for PD, but protective effects of smoking and coffee are among the few consistent findings [1]. In the last 35 years, numerous studies have evaluated the association between PD and rural living, well-water consumption, farming and pesticide use [2], and interest has focused on the quaternary ammonium herbicide paraquat (PQ) in part because of its structural similarity to 1-methyl-4-phenylpyridine (MPP+), a metabolite of 1-methyl-4-phenyl-1,2,3,6-tetrahydropyridine (MPTP). Systemic exposure to MPTP has been shown to cause permanent parkinsonism in humans, non-human primates and rodents as a result of its ability to cross the blood-brain barrier and cause toxicity after being metabolised to MPP+ [3]. Many experimental animal and in vitro studies of PQ have been performed looking for effects such as deficits in dopaminergic neurons, but there remain many challenges in interpreting the relevance of these studies to humans [4].

A mortality study of workers who were engaged in the manufacture of PQ at Widnes in the northwest of England is one of the few cohort studies of PD and exposure to PQ [5]. A key strength of this study was that exposure to PQ was confirmed by comprehensive job histories and the availability of personal monitoring information. At the time that the study was performed, most of the epidemiological studies providing information about a possible association between PD and exposure to PQ were case-control studies with small numbers of subjects exposed to PQ and/or limited exposure information, and the evidence available from them was described as fragmentary and insufficient to establish whether herbicides, and PQ in particular, increase the risk for PD. [3] A possible association between PD and exposure to PQ has continued to be debated, and summary estimates of the relative risk (RR) have been derived in at least four meta-analyses [2, 6–8], and a review and evaluation of several key epidemiological studies of PQ and PD has also been performed [9]. The meta-analyses have all reported a statistically significant association between PQ exposure and PD, but the most recent [8] noted that objective measurement of PQ exposure was inadequate in the studies that were summarised, and concluded that further studies to elucidate the effect of PQ on PD are still warranted.

A cohort of PQ production workers at the Widnes site was originally assembled in the late 1970s as part of an investigation conducted when some of them were found to be suffering from skin lesions including solar keratosis, squamous cell carcinoma, and Bowen’s disease [10]. The investigation concluded that exposure to tarry by-products was the most likely cause of the skin lesions. A retrospective mortality study was later conducted of an extended cohort that included all employees engaged in PQ production at the Widnes site between 1961, when production commenced, and 1983, with follow-up through 31 December 1985 [11], but did not identify any exposure-related health effects. A modest excess of lung cancer (13 observed, 10.5 expected deaths) was reported, but it was concluded that this was unlikely to be occupational in nature. The most recent update included all workers employed up to 1995 when PQ production ceased at the site, with follow-up through 30 June 2009, and confirmed the absence of exposure-related health effects including PD, and reported reduced mortality from lung cancer, especially compared to local mortality rates [5]. The excess originally reported appeared to have been confined to a small group of process workers with less than 1 year of exposure during the period 15 to 30 years after first exposure, but no further lung cancer deaths had occurred among these workers.

This report describes an update of the study of PQ production workers [5] with follow-up extended to 31 December 2017. The primary objective of the study was to assess whether there is any evidence of increased PD mortality as the underlying cause of death or as a mentioned cause of death. A secondary objective was to provide updated information on mortality from major causes of death to confirm the absence of other exposure related effects.

## Methods

### Study population and follow up

The investigation is an update of a retrospective cohort mortality study of all employees who had ever worked on any of the plants at Widnes, UK where PQ was manufactured between 1961 and 1995 (926 male and 42 female workers) [5]. Four plants using different processes were used to manufacture PQ at the site: a high temperature sodium (HTS) plant (from 1961 to 1969, but used only for quaternisation from early 1964); a magnesium (MAG) plant (1962 to 1967); a low temperature sodium (LTS) plant (1966 until 1995 when manufacture of PQ at Widnes ceased); and a plant utilising an ammonia cyanide (AC) process (1985 to 1993). Small scale pre-production versions of the MAG and LTS plants were also operated on the site for short periods.

The vital status of the cohort on 31 December 2017 and cause of death information was obtained from NHS Digital. The underlying cause of death and other causes of death mentioned on the death certificate were coded to the contemporaneous revision of the International Classification of Diseases (ICD).

### Exposure assessment

The previous study report [5] noted that 1330 static monitoring results were collected at Widnes between 1979 and 1993, but only summary information was available for results collected before 1987. In addition, 100 personal monitoring results were collected between 1983 and 1993, but there was insufficient sampling information available to perform a quantitative exposure assessment. A limited qualitative exposure assessment of male workers based on their highest level of exposure to 11 substances including PQ and its manufacturing precursor 4,4′-bipyridyl was also available. Approximately 300 of the 729 male workers included in the initial mortality investigation [11] were assessed in the mid-1980s to have had high or medium exposure to PQ. These included engineering maintenance workers on the MAG and LTS plants, and process operators and plant supervisors on all plants. Workers recruited after this time were unlikely to have experienced exposures to PQ that would have been categorised as high or medium. Exposure levels were not assessed for research staff, plant laboratory workers (day and shift) and technical administrative staff (day and shift), but their exposure was likely to have been low.

### Statistical methods

The observed number of deaths from selected causes and groups of causes was compared with the expected number calculated on the basis of national (England and Wales) and local age and period-specific mortality rates. The local comparison was made using mortality rates available between 1981 and 2017 for the area consisting of Halton unitary authority (where the plants were located) and the 5 surrounding local authorities (district and unitary), and England and Wales mortality rates for the time period 1960–1980. The standardised mortality ratio (SMR) was calculated as the ratio of the observed to the expected deaths. OCMAP-PLUS [12] was used to sum person-years within categories of age (5 year intervals) and calendar period (about 5 year intervals to conform with changes in the ICD), and to compute SMRs and their 95% confidence intervals. Female workers were not included in the SMR analyses because their numbers were small, but their cause of death information was reviewed.

Mortality rates were also calculated for PD using all certified causes of death listed on the death certificate (conventionally termed ‘mentions’), as well as rates for PD as the underlying cause of death. Information on ‘mentions’ was only available for the period 1993–2017. A conservative estimate of the number of deceased workers whose death certificate would be expected to mention PD was calculated using mortality rates for PD as an underlying cause of death to 1992, and a mentioned cause of death after 1992.

Mortality due to PD was also examined in the sub cohort of workers who were assessed to have had high or medium exposure in the original mortality investigation. Duration of employment and time since first exposure were treated as time related variables with the values calculated for each person-year under observation. A subject was allowed to contribute to more than one stratum in each analysis.

## Results

Full details of the plants where male subjects worked are given in the previous report [5]. Over 40% had worked on the two earliest plants (HTS and MAG) and almost half had only worked on the LTS plant. A total of 117 workers were assessed to have held jobs that entailed high exposure to PQ and a further 202 held jobs that entailed medium exposure to PQ. Table 1 shows the vital status of the cohort on 31 December 2017. The average age of male employees at first exposure was 32.8 years and they contributed 33,805 person-years of follow up.

**Table 1:** Vital status on 31 December 2017

Vital Status	Males	Females
Alive	515	20
Dead	394	21
Emigrated or joined armed forces	14	–
Lost to follow up	3	1
Total	926	42

Table 2 shows SMRs for the major causes of death and those of initial interest. Local mortality rates for PD were very similar to England and Wales mortality rates, and only comparisons with England and Wales mortality rates are described in the text. Among male workers, there were two deaths from PD as the underlying cause (3.3 expected), and the death certificates of two other workers mentioned PD (one died of bronchopneumonia and the other of Alzheimer’s disease). At least 6 death certificates of male workers would have been expected to have mentioned PD (SMR = 0.67; 95% CI 0.18–1.72). There was no relationship with duration or level of exposure. Only one of the deceased workers that was exposed for more than 5 years had a death certificate that mentioned PD (2.38 expected mentions). Two of the deceased workers with high or medium PQ-exposure had a death certificate that mentioned PD (2.44 expected mentions) and one of these was exposed for more than 5 years (1.14 expected mentions). None of the death certificates of the 21 deceased female workers mentioned PD. Mortality from all neurological diseases (SMR = 0.44, 5 deaths) was low and there were no mentions of secondary PD disease and other movement disorders on the death certificates of male and female employees.

**Table 2:** Observed numbers of deaths and SMR for selected causes of death among males

***ICD-9***	***Cause of death category***	Observed	England & Wales mortality rates		Local mortality rates^**a**^	
			SMR	95% CI	SMR	95% CI
001–999	All causes of death	394	0.88*	0.80–0.97	0.77**	0.69–0.85
140–208	All malignant neoplasms	136	0.99	0.83–1.17	0.86	0.72–1.01
160–165	Respiratory system	41	0.98	0.71–1.33	0.77	0.55–1.04
162	Bronchus, trachea and lung	38	0.95	0.68–1.31	0.74	0.52–1.02
320–359	Neurological diseases	5	0.44	0.14–1.03	0.43*	0.14–1.00
332.0	Parkinson’s disease	2	0.60	0.07–2.17	0.67	0.08–2.43
332.0	Parkinson’s disease (mentioned)^b^	4	0.67	0.18–1.72	0.68	0.19–1.75
390–398, 402, 404, 410–429	All heart disease	105	0.79*	0.65–0.96	0.70**	0.57–0.88
430–438	Cerebrovascular disease	29	0.92	0.61–1.31	0.84	0.56–1.20
460–519	Non-malignant respiratory disease	50	0.94	0.69–1.23	0.74*	0.55–0.98
800–999	External causes of death	18	1.05	0.62–1.65	0.96	0.57–1.52

* *p* < 0.05, ** *p* < 0.01; SMR significantly different from 1.0

^a^ Halton unitary authority and the 5 surrounding local authorities (district and unitary)

^b^ Mentioned cause of death (1993–2017), underlying cause of death (1960–1992)

Deaths from all causes of death and deaths due to heart disease were significantly lower than expected when compared to England and Wales mortality rates (*p* < 0.05) and local rates (*p* < 0.01). Mortality from non-malignant respiratory disease was slightly less than expected based on England and Wales mortality rates, but significantly lower than expected compared to local rates (*p* < 0.05). A similar pattern was observed for lung cancer mortality, and there was reduced mortality when compared to local mortality rates (SMR = 0.74; 95% CI 0.52–1.02). Mortality from all causes of death was lower during the first 15 years from start of exposure, but there was little relationship with duration of exposure (see supplementary Tables S1 and S2).

In addition, to the 10 causes of death shown in Table 2, comparisons with England and Wales mortality rates were also made for a further 52 causes of death. Among causes with more than 4 expected deaths, SMR > 1 were only observed for cancers of the stomach (SMR = 1.69; 95% CI 0.90–2.90), bladder (SMR = 1.28; 95% CI 0.52–2.64) and prostate (SMR = 1.17; 95% CI 0.67–1.90), and cirrhosis of the liver (SMR = 1.35; 95% CI 0.54–2.78), .and deaths from none of the 52 causes were significantly higher than expected at a *p* = 0.1 level except for stomach cancer (*p* = 0.098). However, deaths from cancers of the colon and rectum were significantly lower than expected (SMR = 0.48; 95% CI 0.19–0.99). Mortality from nephritis and nephrosis was of interest because of the acute renal toxicity of PQ and a hypothesis that exposure to certain pesticides including PQ may increase the risk of end-stage renal disease [13], but no deaths were observed (2.5 expected). Some workers were potentially exposed to benzene, but mortality from lymphohaematopoietic cancers was close to expected (SMR = 0.86; 95% CI 0.39–1.43, 9 observed deaths).

Mortality patterns were also examined among the group of 319 workers who had ever held a job entailing high or medium exposure to PQ. Most of these workers started work on PQ production plants in the 1960s and 204 of them had died by the end of follow up. Compared to local mortality rates, all-cause mortality was lower than expected (SMR = 0.93; 95% CI 0.81–1.07), but deaths due to malignant neoplasms were higher than expected (SMR = 1.08; 95% CI 0.85–1.36), although there was no evidence of any trends with duration of exposure (< 1 year, 1 to 5 years, > 5 years). Both deaths due to PD occurred in this group of workers, but these were the only mentions of PD, and there were fewer PD mentions than expected (SMR = 0.82; 95% CI 0.10–2.97).

## Discussion

Like the earlier investigation [5], the updated study has shown no evidence of increased mortality (underlying and mentioned cause) from PD among PQ production workers. Since the previous investigation, summary estimates of the relative risk (RR) of PD due to PQ exposure have been derived in at least four meta-analyses [2, 6–8]. However, most of the epidemiological information about a possible association between PD and PQ exposure is still provided by case-control studies. Many of these had potential for recall bias, small numbers of subjects exposed to PQ and/or limited exposure information, and few were able to control for confounding. The most recent meta-analysis [8] identified 22 case-control studies and one cohort study, but 9 of the case-control studies were excluded from the meta-analysis because they shared participants with an included study. A random effects summary OR of 1.64 (95% CI 1.27–2.13) was calculated for the 13 case-control studies. However, the investigators only identified 3 studies that had reported an adjusted odds ratio (OR), and they calculated crude ORs for 8 studies including two early case-control studies for which ORs were not reported because each had only a single exposed case and no exposed controls [14, 15], and three studies which had reported more appropriate ORs based on matched or adjusted analyses [16–18]. Furthermore, the crude OR reported by another study [19] was replaced by a much larger, but incorrectly calculated, crude OR. The meta-analysis included only one case-control study [18] published after the cut-off date for an earlier meta-analysis [2] which derived a random effects summary OR of 1.47 (95% CI 1.27–2.13), and which was also based on risk estimates from 13 case-control studies. The studies included by the two meta-analyses were broadly similar, but the earlier one [2] did not include findings from the two early case-control studies [14, 15] which had no exposed controls. However, risk estimates from two studies not cited by the most recent meta-analysis [8] were included: the study of PQ manufacturing workers [5] and a parkinsonism prevalence study [20], although the ratio of PD cases to parkinsonism cases (1:65) in the latter study was very low and its investigators noted that most subjects with parkinsonism showed only slight signs of parkinsonism and there was possible over ascertainment of rigidity. In addition, the earlier meta-analysis [2] included the OR for incident cases from a study [21] which was excluded by the most recent meta-analysis [8] because of overlap with the incident and prevalent cases of another study [22]. Two earlier meta-analyses [6, 7] conducted at a similar time gave contrasting results. One [6] derived a fixed effects summary OR of 2.19 (95% CI 1.48–3.26) for 7 studies which reduced to 1.72 (95% CI 1.28–2.32) when the two studies of lowest quality were excluded, and the other [7] derived a fixed effects summary OR of 1.32 (96% CI 1.10–1.60) for 9 studies.

The most recent meta-analysis [8] also concluded that two studies [16, 22] were able to link longer exposures with PD. However, the ORs from one study [22] that were claimed to show a PD risk that increases with 5, 10, and 15 years of exposure were in fact ORs for the association between ever use of PQ and PD for exposure truncated at 5, 10, or 15 years before the diagnosis or reference date.

A recent French cohort study of agricultural workers [23] was not included in the most recent meta-analysis [8], but was discussed in its systematic review. This large study of over 180,000 participants included 1732 subjects (1.2%) who reported PD at enrolment in the cohort. In models adjusted for age, sex, educational level, smoking status and alcohol consumption, significantly elevated ORs of a similar magnitude were reported for all 14 pesticide active ingredients of a priori interest, including PQ. However, when a term for exposure to another active ingredient was added to the model, only the ORs for zineb and ziram remained significantly elevated, and the OR for PQ reduced from 1.43 (95% CI 1.17–1.75) to 1.01 (95% CI 0.41–2.49). There was also no association with duration of PQ use whichever model was used.

The present study of workers engaged in the manufacture of PQ has the advantage of being a cohort study, with no potential for recall bias. In addition, comprehensive job histories enable workers with the highest potential for exposure to be identified, whereas most of the PQ-specific studies have no satisfactory information about definition, duration and extent of exposure.

The study has limited power to detect a risk estimate as low as that suggested by the more recent meta-analyses, although the upper confidence limit of the SMR for mentions of PD is relatively low (1.72). However, a strength of the study is the likely higher exposure of workers engaged in PQ production than many subjects in case-control studies classified as exposed to PQ. Limited information is available about exposure conditions and personal protective equipment (PPE) use in the 1960s when there was less understanding about the effect of dermal or inhalation exposure [24]. A document describing handling precautions during manufacture at Widnes in the early 1970s noted that ingestion of a high dose of PQ led to liver or kidney failure within 2 or 3 days, but that smaller doses could result in a progressive pulmonary insufficiency leading to death. The presence of dust particles was considered to be the major hazard in manufacture and formulation of PQ, and it was noted that inhalation of dust particles causes nose bleeding which ceases on removal from exposure. It was stated that the dust hazard can be reduced by careful attention to cleanliness and avoidance of spillages at all stages of the operation, and lists the PPE that should be worn for some operations. This included a full face respirator to trap dust during plant maintenance and quaternisation. Many of the workers with skin lesions reported nose bleeds when interviewed as part of the investigation described earlier [10], as did most workers handling solid formulations of PQ at another plant who wore partial PPE including an approved dust mask [25]. The prevalence of nose bleeds was considerably higher than that seen in cross-sectional surveys of PQ sprayers. For instance, a study of Sri Lankan sprayers reported that only 3 of 85 sprayers reported nose bleeds [26].

Personal monitoring results collected after 1983 when all jobs were assessed as having low exposure, were well below the UK occupational exposure limit of 0.08 mg/m^3^ (8 h TWA, respirable fraction). The geometric mean of 0.003 mg/m^3^ equates to a mean daily intake of 25.8 mg PQ ion. This value is not directly comparable with reported levels of PQ excreted over 24 h in the urine of PQ sprayers. However, as the predominant route of excretion is via urine, it was concluded that the exposure of a PQ production worker on a daily basis is at least comparable with that of a PQ sprayer [5], especially for jobs assessed as having high or medium exposure since exposure levels were almost certainly higher during earlier years of production. In addition, workers engaged in PQ production had the potential to be exposed on a daily basis over many years, whereas most workers employed as sprayers are unlikely to have sprayed PQ on a daily basis. Many subjects described as exposed to PQ in the published case-control studies may only have used PQ on a very occasional basis, or have been inferred to have had ambient exposure.

Another limitation of this study is that cases of PD are only identified if PD is a certified cause of death listed on their death certificate. However, although it is widely regarded that most patients with PD die of its complications and not the disease, the limited information available suggests that PD is coded as the underlying cause of death of many patients and is mentioned on the death certificates of the majority of patients. Four UK studies reported figures of 53% [27], 60% [28], 63% [29], and 76% [30] for the percentage of people with PD whose death certificates listed PD in either part I or part II. One of these studies also reported that PD was coded as the underlying cause of death of 37% of death certificates [30]. These studies suggest that less than 4 of the deceased workers in the present study would be expected to have had PD which was not mentioned on their death certificates. More importantly, the SMR is not biased as the same degree of underreporting applies to national and local mortality rates.

The feasibility of conducting a morbidity study of the whole group was also considered at the time of the earlier study, but it was decided that performing a morbidity study would not be worthwhile unless the results of the mortality investigation indicated an increased risk of PD.

Another limitation of the study is that rates for mentions of PD on death certificates could only be calculated from 1993 onwards and the analysis of mentions of PD had to use underlying cause of death rates before 1993. However, this resulted in an underestimate of the number of expected mentions of PD. Two UK studies provide some information about trends in mentions of PD on death certificates before 1993 [31, 32], and suggest that underlying cause PD deaths between 1985 and 1993 (0.4 expected deaths) would have been about half of all deaths with mentions of PD. Very few deaths due to PD were expected before 1985, and consequently the true expected number of PD mentions over the study period is likely to have been underestimated by ~ 0.4 mentions.

Smoking histories were not available for the workers, and smoking is a protective factor for PD. However, mortality from non-malignant respiratory disease was significantly reduced compared to the local population, and does not suggest a higher smoking prevalence in the workforce. Limited information was collected about work history before entering the cohort, but this was unlikely to be relevant to the primary aim of the study. Many of the earliest entrants to the cohort were experienced workers who transferred to the PQ production plants from other plants at the Widnes site which produced a number of chemical compounds including a variety of pesticides. Many later recruits were company employees who moved from one of the large company production sites in the vicinity.

A full quantitative exposure assessment was not conducted. As noted earlier, no quantitative information about PQ exposure was available before 1979, but the report of the qualitative exposure assessment performed in the mid-1980s noted that exposures to PQ on the LTS plant were much lower then than during the 1960s. Three of the workers whose death certificates mentioned PD were maintenance workers who did not work permanently on the PQ production plants and who would only have worked there occasionally or during shutdown periods. In the case of PD, the absence of additional quantitative exposure information was not a limitation as there were only four mentions of PD on the death certificates of deceased workers, and hence limited information to assess exposure response. However, the SMR for PD mentions of 0.82 for the group with high and medium exposure to PQ was slightly higher than that for all workers (SMR = 0.67).

## Conclusions

There was no evidence of an increased incidence of PD among PQ production workers based on mentions of PD on the death certificates of workers who had died. In addition, there was no evidence of adverse mortality due to other causes including lung cancer mortality.

## Supplementary Information

**Additional file 1: Table S1.** Observed numbers of deaths and SMR for selected causes of death among males by time since first exposure. **Table S2.** Observed numbers of deaths and SMR for selected causes of death among males by duration of exposure.

## Data Availability

Pseudonymised outcome data were supplied by NHS Digital under a data sharing agreement subject to there being no risk of identifying any individual in the publication. However, as our cohort data contains detailed information on each workers job-history and health outcomes, identification of an individual is a possibility if raw data would be freely available. Derived data from this cohort is available in our repository by writing to the corresponding author, but researchers who would like to use the data will need to obtain agreement from NHS Digital.

## Abbreviations

AC: Ammonia cyanide
CI: Confidence interval
HTS: High temperature sodium
ICD: International Classification of Diseases
LTS: Low temperature sodium
MAG: Magnesium
OR: Odds ratio
PD: Parkinson’s disease
PQ: Paraquat
RR: Relative risk
SMR: Standardised mortality ratio

## References

[1] Wirdefeldt K, Adami HO, Cole P, Trichopoulos D, Mandel J (2011). Epidemiology and etiology of Parkinson's disease: a review of the evidence. Eur J Epidemiol.

[2] Breckenridge CB, Berry C, Chang ET, Sielken RL, Mandel JS (2016). Association between Parkinson’s disease and cigarette smoking, rural living, well-water consumption, farming and pesticide use: systematic review and meta-analysis. PLoS One.

[3] Berry C, La Vecchia C, Nicotera P (2010). Paraquat and Parkinson's disease. Cell Death Differ.

[4] Boyd WA, Blain RB, Skuce CR, Thayer KA, Rooney AA (2020). NTP research report on the scoping review of paraquat dichloride exposure and Parkinson’s disease.

[5] Tomenson JA, Campbell C (2011). Mortality from Parkinson’s disease and other causes among a workforce manufacturing paraquat: a retrospective cohort study. BMJ Open.

[6] Pezzoli G, Cereda E (2013). Exposure to pesticides or solvents and risk of Parkinson disease. Neurology..

[7] Ntzani EE, Chondrogiorgi M, Ntritsos G, Evangelou E, Tzoulaki I (2013). Literature review on epidemiological studies linking exposure to pesticides and health effects. EFSA supporting publication.

[8] Tangamornsuksan W, Lohitnavy O, Sruamsiri R, Chaiyakunapruk N, Norman Scholfield C, Reisfeld B, Lohitnavy M. Paraquat exposure and Parkinson's disease: A systematic review and meta-analysis. Arch Environ Occup Health. 2018. doi:10.1080/19338244.2018.1492894. Erratum in: Arch Environ Occup Health. 2018. doi:10.1080/19338244.2018.1558737. 10.1080/19338244.2018.1492894 30474499

[9] Mandel JS, Adami HO, Cole P (2012). Paraquat and Parkinson's disease: an overview of the epidemiology and a review of two recent studies. Regul Toxicol Pharmacol.

[10] Bowra GT, Duffield DP, Osborn AJ, Purchase IFH (1982). Premalignant and neoplastic skin lesions associated with occupational exposure to "tarry" by-products during manufacture of 4,4'-bipyridyl. Br J Ind Med.

[11] Paddle GM, Osborn AJ, Parker GD (1991). Mortality of employees in plants manufacturing 4,4′-bipyridyl. Scand J Work Environ Health.

[12] Marsh GM, Youk AO, Stone RA, Sefcik S, Alcorn C (1998). OCMAP-PLUS: a program for the comprehensive analysis of occupational cohort data. J Occup Environ Med.

[13] Lebov JF, Engel LS, Richardson D, Hogan SL, Hoppin JA, Sandler DP (2016). Pesticide use and risk of end-stage renal disease among licensed pesticide applicators in the agricultural health study. Occup Environ Med.

[14] Semchuk KM, Love EJ, Lee RG (1992). Parkinson's disease and exposure to agricultural work and pesticide chemicals. Neurology..

[15] Seidler A, Hellenbrand W, Robra B-P, Vieregge P, Nischan P, Joerg J, Oertel WH, Ulm G, Schneider E (1996). Possible environmental, occupational, and other etiologic factors for Parkinson's disease: a case-control study in Germany. Neurology..

[16] Liou HH, Tsai MC, Chen CJ, Jeng JS, Chang YC, Chen SY, Chen RC (1997). Environmental risk factors and Parkinson’s disease: a case-control study in Taiwan. Neurology..

[17] Firestone JA, Lundin JI, Powers KM, Smith-Weller T, Franklin GM, Swanson PD, Longstreth WT, Checkoway H (2010). Occupational factors and risk of Parkinson's disease: a population-based case-control study. Am J Ind Med.

[18] Van der Mark M, Vermeulen R, Nijssen PC, Mulleners WM, Sas AM, van Laar T, Brouwer M, Huss A, Kromhout H (2014). Occupational exposure to pesticides and endotoxin and Parkinson disease in the Netherlands. Occup Environ Med.

[19] Hertzman C, Wiens M, Snow B, Kelly S, Calne D (1994). A case-control study of Parkinson's disease in a horticultural region of British Columbia. Mov Disord.

[20] Engel LS, Checkoway H, Keifer MC, Seixas NS, Longstreth WT Jr, Scott KC, Hudnell K, Anger WK, Camicioli R (2001). Parkinsonism and occupational exposure to pesticides. Occup Environ Med.

[21] Kamel F, Tanner C, Umbach D, Hoppin J, Alavanja M, Blair A, Comyns K, Goldman S, Korell M, Langston J, Ross G, Sandler D (2007). Pesticide exposure and self-reported Parkinson’s disease in the agricultural health study. Am J Epidemiol.

[22] Tanner CM, Kamel F, Ross GW, Hoppin JA, Goldman SM, Korell M, Marras C, Bhudhikanok GS, Kasten M, Chade AR, Comyns K, Richards MB, Meng C, Priestley B, Fernandez HH, Cambi F, Umbach DM, Blair A, Sandler DP, Langston JW (2011). Rotenone, paraquat, and Parkinson's disease. Environ Health Perspect.

[23] Pouchieu C, Piel C, Carles C, Gruber A, Helmer C, Tual S, Marcotullio E, Lebailly P, Baldi I (2018). Pesticide use in agriculture and Parkinson’s disease in the AGRICAN cohort study. Int J Epidemiol.

[24] Swan AA (1969). Exposure of spray operators to paraquat. Br J Ind Med.

[25] Howard JK (1979). A clinical survey of paraquat formulation workers. Br J Ind Med.

[26] Senanayake N, Gurunathan G, Hart TB, Amerasinghe P, Babapulle M, Ellapola SB, Udupihille M, Basanayake V (1993). An epidemiological study of the health of Sri Lankan tea plantation workers associated with long term exposure to paraquat. Br J Ind Med.

[27] Hobson P, Meara J (2018). Mortality and quality of death certification in a cohort of patients with Parkinson's disease and matched controls in North Wales, UK at 18 years: a community-based cohort study. BMJ Open.

[28] Williams-Gray CH, Mason SL, Evans JR, Foltynie T, Brayne C, Robbins TW, Barker RA (2013). The CamPaIGN study of Parkinson's disease: 10-year outlook in an incident population-based cohort. J Neurol Neurosurg Psychiatry.

[29] Pennington S, Snell K, Lee M, Walker R (2010). The cause of death in idiopathic Parkinson's disease. Parkinsonism Relat Disord.

[30] Phillips NJ, Reay J, Martyn CN (1999). Validity of mortality data for Parkinson’s disease. J Epidemiol Community Health.

[31] Griffiths C, Rooney C (2006). Trends in mortality from Alzheimer’s disease, Parkinson’s disease and dementia, England and Wales, 1979-2004. Health Stat Q.

[32] Goldacre MJ, Duncan M, Griffith M, Turner MR (2010). Trends in death certification for multiple sclerosis, motor neuron disease, Parkinson's disease and epilepsy in English populations 1979-2006. J Neurol.

