# Supplementary material for: Mortality from Parkinson’s disease and other causes among a workforce manufacturing paraquat: an updated retrospective cohort study
Source: J Occup Med Toxicol. 2021 May 27;16:20. doi: 10.1186/s12995-021-00309-z (PMC8157632; doi:10.1186/s12995-021-00309-z)
Supplement: Supplementary file 1 — Additional file 1: Table S1. Observed numbers of deaths and SMR for selected causes of death among males by time since first exposure. Table S2. Observed numbers of deaths and SMR for selected causes of death among males by duration of exposure. [file 12995_2021_309_MOESM1_ESM.docx]

**Table S1** Observed numbers of deaths and SMR^1^ for selected causes of death among males by time since first exposure

| ***ICD-9*** | ***Cause of death category*** | **Observed** | **0-15 years** | | **Observed** | **> 15 years** | |
| --- | --- | --- | --- | --- | --- | --- | --- |
|  |  |  | **SMR** | **95% CI** |  | **SMR** | **95% CI** |
| 001-999 | All causes of death | 46 | 0.68** | 0.50 – 0.91 | 348 | 0.78** | 0.70 - 0.87 |
| 140-208 | All malignant neoplasms | 16 | 0.87 | 0.50 – 1.41 | 120 | 0.85 | 0.71 – 1.02 |
| 160-165 | Respiratory system | 4 | 0.54 | 0.15 – 1.38 | 37 | 0.80 | 0.57 – 1.11 |
| 162 | Bronchus, trachea and lung | 4 | 0.56 | 0.15 – 1.44 | 34 | 0.77 | 0.53 – 1.08 |
| 320-359 | Neurological diseases | 0 | - | 0.00 – 3.39 | 5 | 0.47 | 0.15 – 1.10 |
| 332.0 | Parkinson’s disease | 0 | - | 0.00 – 50.6 | 2 | 0.69 | 0.08 – 2.49 |
| 332.0 | Parkinson’s disease (mentioned)^2^ | 0 | - | 0.00 – 50.7 | 4 | 0.69 | 0.19 – 1.77 |
| 390-398, 402, 404, 410-429 | All heart disease | 12 | 0.50* | 0.26 – 0.87 | 93 | 0.74** | 0.60 – 0.91 |
| 430-438 | Cerebrovascular disease | 4 | 0.95 | 0.26 – 2.44 | 25 | 0.82 | 0.53 – 1.21 |
| 460-519 | Non-malignant respiratory disease | 3 | 0.51 | 0.11 – 1.50 | 47 | 0.76 | 0.56 – 1.01 |
| 800-999 | External causes of death | 4 | 0.61 | 0.17 – 1.56 | 14 | 1.15 | 0.63 – 1.93 |

* p < 0.05, ** p < 0.01; SMR significantly different from 1.0

^1^ Local mortality rates for Halton unitary authority and the 5 surrounding local authorities (district and unitary)

^2^ Mentioned cause of death (1993 -2017), underlying cause of death (1960-1992)

**Table S2** Observed numbers of deaths and SMR^1^ for selected causes of death among males by duration of exposure

| ***ICD-9*** | ***Cause of death category*** | **Observed** | **0-1 years** | | **Observed** | **1-5 years** | | **Observed** | **> 5 years** | |
| --- | --- | --- | --- | --- | --- | --- | --- | --- | --- | --- |
|  |  |  | **SMR** | **95% CI** |  | **SMR** | **95% CI** |  | **SMR** | **95% CI** |
| 001-999 | All causes of death | 93 | 0.84 | 0.67 – 1.02 | 132 | 0.66** | 0.56 - 0.79 | 169 | 0.85* | 0.72 - 0.98 |
| 140-208 | All malignant neoplasms | 35 | 1.03 | 0.72 – 1.44 | 45 | 0.73* | 0.53 - 0.98 | 56 | 0.89 | 0.67 - 1.15 |
| 160-165 | Respiratory system | 10 | 0.86 | 0.41 – 1.58 | 16 | 0.78 | 0.45 – 1.27 | 15 | 0.71 | 0.40 - 1.18 |
| 162 | Bronchus, trachea and lung | 9 | 0.81 | 0.37 – 1.53 | 14 | 0.71 | 0.39 – 1.19 | 15 | 0.75 | 0.42 – 1.23 |
| 320-359 | Neurological diseases | 0 | - | 0.00 – 1.51 | 2 | 0.43 | 0.05 – 1.54 | 3 | 0.65 | 0.13 – 1.90 |
| 332.0 | Parkinson’s disease | 0 | - | 0.00 – 6.03 | 1 | 0.85 | 0.02 – 4.75 | 1 | 0.82 | 0.02 – 4.59 |
| 332.0 | Parkinson’s disease (mentioned)^2^ | 0 | - | 0.00 – 3.19 | 3 | 1.30 | 0.27 – 3.81 | 1 | 0.42 | 0.01 – 2.33 |
| 390-398, 402, 404, 410-429 | All heart disease | 24 | 0.73 | 0.47 – 1.08 | 29 | 0.51** | 0.34 – 0.73 | 52 | 0.89 | 0.67 – 1.17 |
| 430-438 | Cerebrovascular disease | 7 | 0.91 | 0.37 – 1.88 | 9 | 0.68 | 0.31 – 1.28 | 13 | 0.96 | 0.51 – 1.65 |
| 460-519 | Non-malignant respiratory disease | 13 | 0.90 | 0.48 – 1.54 | 17 | 0.65 | 0.38 – 1.04 | 20 | 0.76 | 0.47 – 1.18 |
| 800-999 | External causes of death | 4 | 0.86 | 0.23 – 2.19 | 9 | 1.16 | 0.53 – 2.20 | 5 | 0.79 | 0.26 – 1.84 |

* p < 0.05, ** p < 0.01; SMR significantly different from 1.0

^1^ Local mortality rates for Halton unitary authority and the 5 surrounding local authorities (district and unitary)

^2^ Mentioned cause of death (1993 -2017), underlying cause of death (1960-1992)
